# Determinants of coexistence of undernutrition and anemia among under-five children in Rwanda; evidence from 2019/20 demographic health survey: Application of bivariate binary logistic regression model

**DOI:** 10.1371/journal.pone.0290111

**Published:** 2024-04-05

**Authors:** Abebew Aklog Asmare, Yitateku Adugna Agmas

**Affiliations:** 1 Department of Statistics, Mekdela Amba University, Tuluawlyia, Ethiopia; 2 Department of Rural Development and Agricultural Extension, Mekdela Amba University, Tuluawlyia, Ethiopia; University of Gour Banga, INDIA

## Abstract

**Background:**

Undernutrition and anemia are significant public health issues among under-5 children, with potential long-term consequences for growth, development, and overall health. Thus, this study aims to conduct a bivariate binary logistic regression model by accounting for the possible dependency of childhood undernutrition and anemia.

**Methods:**

The data came from the DHS program’s measurement. A total of 3,206 under-five children were involved in this study. A single composite index measure was calculated for stunting, wasting, and underweight using principal component analysis. A bivariate binary logistic regression model is used to assess the association between undernutrition and anemia given the effect of other predictors.

**Results:**

Among 3,206 under-five children considered in this study, 1482 (46.2%) and 658 (20.5%) children were agonized by anemia and undernutrition, respectively. In bivariate binary logistic regression model; Urban children [AOR = 0.751, 96% CI: 0.573–0.984; AOR = 0.663, 95% CI: 0.456–0.995] and anemic mothers [AOR = 1.160, 95% CI: 1.104–1.218; AOR = 1.663, 95% CI: 1.242–2.225] were significantly associated with both childhood anemia and undernutrition, respectively. Improved water sources [AOR = 0.681, 95% CI: 0.446–0.996], average-sized children [AOR = 0.567, 95% CI: 0.462–0.696], and diarrhea [AOR = 1.134, 95% CI: 1.120–2.792] were significantly associated with childhood anemia. Large-sized children [AOR = 0.882, 95% CI: 0.791–0.853] and those with fever [AOR = 1.152, 95% CI: 1.312–2.981] were significantly associated with under-five children’s undernutrition.

**Conclusion:**

The prevalence of both undernutrition and anemia among under-five-year-old children was high in Rwanda. The following determinants are statistically associated with both childhood undernutrition and anemia: place of residence; source of drinking water; maternal anemia; being a twin; birth size of children; diarrhea; fever; and child age. Anemia and nutritional deficiencies must be treated concurrently under one program, with evidence-based policies aimed at vulnerable populations.

## Introduction

Undernutrition and anemia are serious public health problems that can have long-term effects on a child’s growth, development, and general health [1]. World health organization [2] reports that in 2017, the prevalence of anemia in children under the age of five was 41.7% worldwide. As a result, the issue is made worse in Africa, where anemia affects more than 59% of children under the age of five [3]. According to the United Nations Children’s Fund [4], 22% of children under the age of five were stunted, 12.6% were underweight, and 6.7% were wasted in 2020. In 2020, undernutrition will be the leading cause of death for children under the age of five globally.

Rwanda has endured a civil war, a genocide, and the subsequent rehabilitation of the nation over the past 25 years (1992–2017). Positive trends in the decline of undernutrition have been observed in Rwanda during the reconstruction phase. Children under the age of five who were underweight fell from 15% to 8% between 2005 and 2019/20 [5, 6]. Additionally, the percentage of stunts has dropped from 51% in 2005 to 33% in 2019–20 [5, 6]. Similarly, between the ages of 6 months and 5 years, the prevalence of anemia has dropped from 51% to 37% [5, 6]. Despite these advancements, the high frequency of underweight, stunting, and anemia continues to be a public health problem. The most important health problems affecting Rwandan children are still anemia and malnutrition, especially among children under the age of five, who are particularly susceptible to anemia and malnutrition [7, 8]. Additionally, despite the fact that undernutrition and anemia may be declining nationally, the shift has not been consistent nationwide. Instead, there has been variation in the prevalence of anemia and stunting as well as the rates of change in anemia and stunting across the 30 distinct districts of the nation [9]. The Rwandan government has, in recent years, consistently increased its commitment to nutrition and implemented a number of measures to address these issues both directly and through measures to address the fundamental and underlying causes of malnutrition. Anemia and child malnutrition, however, are still significant public health problems in Rwanda [9].

Stunting shows a substantial correlation with child age, wealth index, maternal education, and the number of antenatal care visits, according to a Rwandan study on children under the age of five [10]. The same study in Rwanda on children under the age of five found a strong correlation between anemia and parental education level, geographic location, and the child’s sex [11]. According to studies [8, 12–16], there are numerous factors that contribute to child malnutrition and anemia. Among the prevalent determinants are socioeconomic inequality, feeding habits, geographic disparities, household food instability, and mother literacy.

A study done in Ethiopia found that the common and significant predictors of stunting and underweight included the child’s age, anemia level, and type of birth. A child’s birth size was strongly linked to a higher risk of stunting, underweight, and wasting in children under the age of five [17]. Significant correlations have been shown between undernutrition and region, fever in the previous two weeks, birth type, mother’s BMI, parents’ education level, wealth index, family size, number of children, and maternal age [13–15]. The study conducted in Rwanda revealed that the child’s age, the duration of breastfeeding, the gender of the child, the nutritional status of the child (whether underweight and/or wasting), whether the child had a fever or had a cough in the two weeks prior to the survey or not, whether the child received vitamin A supplementation in the six weeks before the survey or not, the household wealth index, the literacy of the mother, the mother’s anemia status, mother’s age at birth are all significant factors associated with childhood anemia [8].

Studies on anemia and undernutrition have been conducted in many countries, including Nigeria [18], Ethiopia [18], Gambia [12], and Rwanda [7, 8, 19–21]. However, there is a dearth of literature and little focus on their relationship. It has long been understood that anemia and malnutrition in children have negative, long-term repercussions on human advancement [22]. They frequently co-occur in the same populations and with the same kids because they are closely related. Both anemia and undernutrition are linked to higher death rates, particularly when both conditions are present in the same child [23]. The high prevalence of anemia and malnutrition in children under the age of five raises the possibility that the two diseases may be related. Therefore, a thorough investigation is needed to ascertain whether and how children’s nutritional status is linked to their anemic status, as well as whether the association is affected by additional risk factors. Although some studies [24, 25] used separate analyses of anemia and undernutrition data using binary and ordinal logistic regression to try to find common factors associated with anemia and undernutrition indicators (measured by stunting, underweight, and wasting), such an analysis may be ineffective in determining whether there is any association between undernutrition and anemia. In contrast to this context, the current study analyzes data from the 2019/20 Rwanda Demographic and Health Survey (RDHS) and bivariate binary logistic regression models to examine the association between anemia and undernutrition in under-five children. With the help of this study, we hoped to spot potential for accelerating the reduction of anemia and undernutrition in Rwanda. This finding can be utilized to direct upcoming plans and financial commitments for anemia and nutrition in Rwanda. Additionally, these findings might be instructive for other nations with comparable profiles that want to address issues with undernutrition and anemia.

## Description of preprint

A preprint has previously been published [17]. However, the discovery has yet to be published in another journal.

## Materials and methods

### Data source and sampling method

The researchers used data from the 2019/20 Rwanda Demographic and Health Surveys (RDHS) in this study. For some limited indicators, the 2019/20 RDHS used a two-stage sample design and was intended to allow estimates of key indicators at the national level, as well as for urban and rural areas, five provinces, and each of Rwanda’s 30 districts. The first step was to select sample points (clusters) made up of EAs delineated for the 2012 RPHC. A total of 500 clusters were chosen, with 112 in cities and 388 in rural areas. The second stage involved systematic household sampling. From June to August 2019, all selected EAs conducted a household listing operation, and households to be included in the survey were drawn at random from these lists. Each sample point selected 26 households, for a total sample size of 13,000 households. The sample is not self-weighted at the national level due to the approximately equal sample sizes in each district, and weighting factors have been added to the data file so that the results are proportional at the national level [26]. The dependent and independent variables in this study were extracted from the Kid Record (KR file) data set. This study used a weighted total sample of 3,206 children under the age of five. Data was collected from November 9, 2019, to July 20, 2020, and the whole sampling technique was detailed in the comprehensive RDHS report [26].

### Inclusion/exclusion criteria

The inclusion criteria were children aged less than five years who had completed relevant forms about their personal information and clinical signs. Therefore, children who had not acquired all the related information or were older than or equal to five years were left out.

### Study variables and measurements

#### Dependent variables

Three response variables undernutrition and anemia were considered in this paper. The three undernutrition indicators were measured through standardized score (z-score) for height-for-age (stunting), weight-for-height (wasting), and weight-for-age (underweight). Z-score for the *i*^*th*^ child (*Z*_*i*_) is defined as $Z_{i}=\frac{{AI}_{i}-\mu }{\sigma }$, where *AI*_*i*_, *μ and σ* is stunting, underweight, and wasting of the *i*^*th*^ child, median and standard deviation respectively. A single composite index measure was calculated, because the three undernutrition indicators have significant correlation (Table 1) from for height-for-age (stunting), weight-for-height (wasting), and weight-for-age (underweight) using principal component analysis [27, 28]. The first component alone explains 66.5% (Table 1) of the total variation of all undernutrition indices and this amount is significantly high sufficient to produce a sole composite index of undernutrition [29].

**Table 1:** Eigen value, principal component and correlation of the three anthropometric variables.

Eigen value	Proportion	Cumulative	PC	HAZ (Corr)	WAZ (Corr)	WHZ (Corr)
1.995	0.665	0.665	1	0.521 (1)	0.600 (0.42)	0.608 (0.44)
0.631	0.210	0.875	2	0.852	-0.410 ()	-0.325 (0.62)
0.375	0.125	1.000	3	0.054	0.687	-0.725

Corr: correlation; PC: principal competent; HAZ: height for age standard score; WAZ: Weight for age standard score; WHZ: weight for height standard score

Therefore, 0.521*HAZ*+0.600*WAZ*+0.608*WHZ* was taken as a new single composite index of undernutrition which was again classified as nutrition status for each child is calculated, the response variables were recoded into dichotomies response variables as: (0 = Nourished *if Z*−*score*≥−2 *and* 1 = *Under*nourished *if Z*−*score*<−2) according to WHO 2006 child growth standards [30]. Similarly, the occurrence of anemia, based on hemoglobin levels is adjusted for altitude by hemoglobin in grams per deciliter (g/dl) [31]. The anemia status of children aged 6–59 months. For the current analysis, the second response variable (anemia status) was dichotomized indicating not anemic coded by zero or anemic coded by 1 (*not anemic* = 0, *and anemic* = 1).

#### Independent variables

**Fig 1:**
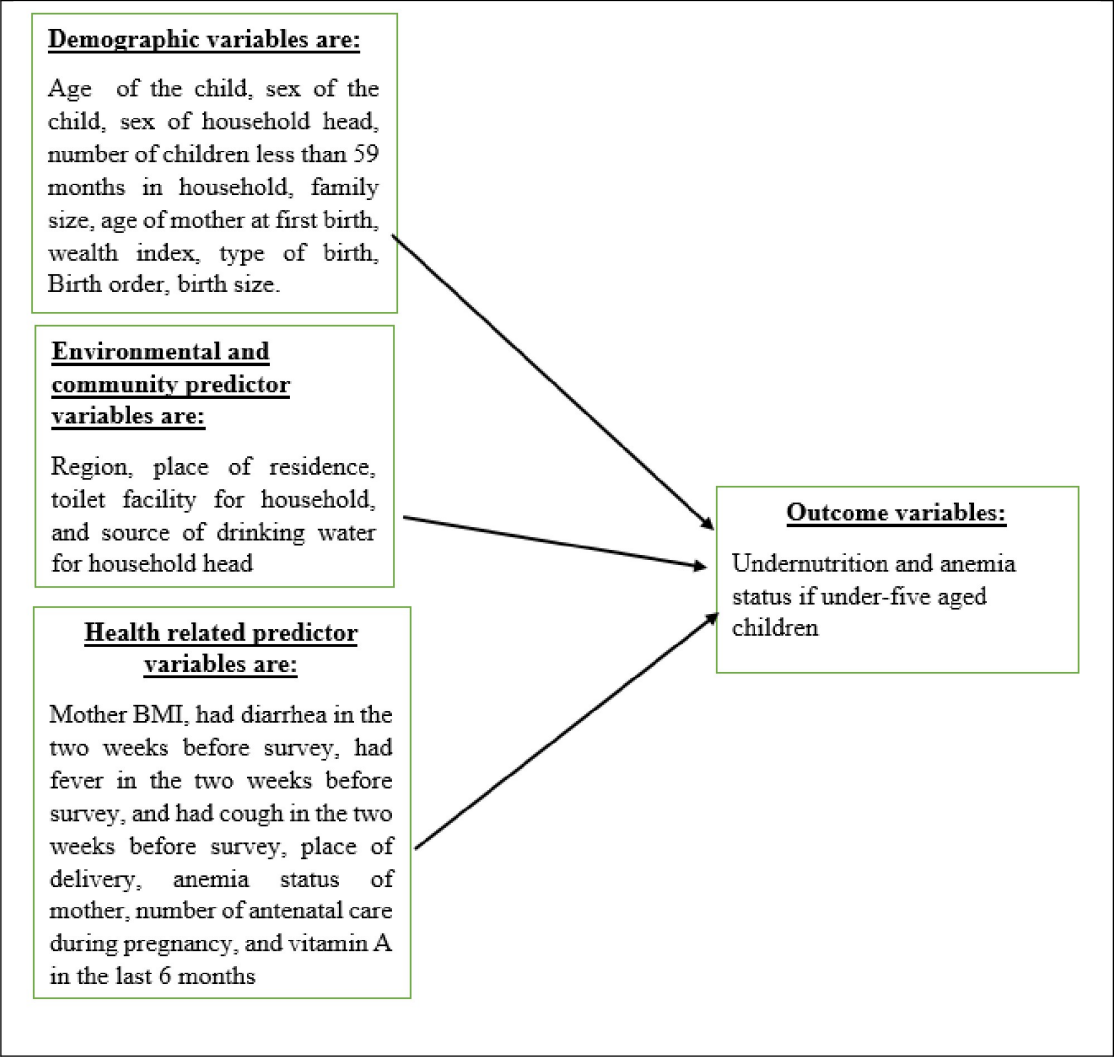
Conceptual framework. Summarizes the methods we used to construct our conceptual framework and how independent variables affect dependent variables.

The independent variables included in this study were region, types of place of residence, highest education level of mother, source of drinking water, religion, number of household members, sex of household head, ethnicity, age of respondent at first birth, husband’s education level, breast feeding, wealth index of household, body mass index of mother, sex of a child, child age in a month, diarrhea, fever, cough, size of children at birth, whether a child is a twin, birth order of a child, number of under-five children in the household, mother anemia level, and child anemia level [7, 8, 12, 20, 32–35]. The above-listed independent variables that are correlated to anemia and undernutrition indicators are presented in Table 3. The parameters in Tables 2 and 3 were collected using face-to-face interviews of mothers ‘or care givers’ (Fig 1).

**Table 2:** Frequency and percentage distribution of the outcome variables.

Outcome variable	Categories (codes)	Frequency (%)
Anemia status	Not anemic	1724 (53.8)
	Anemic	1482 (46.2)
Undernutrition status	Nourished	2548 (79.5)
	Undernourished	658 (20.5)

**Table 3:** Frequency and percentage distribution of the independent variables.

Predictors	Characteristics	Frequency (%)
Region	Kigali	453 (14.1)
	South	621 (19.4)
	West	817 (25.5)
	North	504 (15.7)
	East	811 (25.3)
Type of place of residence	Urban	544 (17.0)
	Rural	2662 (83.0)
Source of drinking water	Not improved	2054 (64.1)
	Improved	1152 (35.9)
Type of toilet facility	Not improved	842 (26.3)
	Improved	2364 (73.7)
Number of household member	1 to 4	1197 (37.3)
	5 to 8	5 to 8 (57.4)
	More than 8	170 (5.3)
Number of children 5 and under in household	Only one	1379 (43.0)
	Two	1490 (46.5)
	3 and more	337 (10.5)
Sex of household head	Male	2747 (85.7)
	Female	459 (14.3)
Wealth index	Poor	1326 (41.4)
	Middle	632 (19.7)
	Rich	1248 (38.9)
Age of respondent at first birth	Less than 20	1002 (31.3)
	20 to 34	2170 (67.7)
	35 to 49	34 (1.1)
Body mass index of mother	Thin	94(2.9)
	Normal	2101 (65.6)
	Over Weight	761 (23.7)
	Obese	250 (7.8)
Anemia status of mother	Not anemic	2827 (88.2)
	Anemic	379 (11.8)
Covered by health insurance	No	579 (18.1)
	Yes	2627 (81.9)
Highest education level	No education	407 (12.7)
	Primary	2079 (64.9)
	Secondary	575 (17.9)
	Higher	144 (4.5)
Husband/partners education level	No education	429 (13.4)
	Primary	21.94 (68.4)
	Secondary	408 (12.7)
	Higher	175 (5.5)
Respondents current working	No	825 (25.7)
	Yes	2381 (74.3)
Birth order number	First	622 (19.4)
	2 to 3	1390 (43.4)
	4 to 5	712 (22.2)
	6 and more	428 (15.0)
Child is twin	Single birth	3116 (97.2)
	Multiple birth	90 (2.8)
Sex of child	Male	1640 (51.2)
	Female	1566 (48.8)
Number of antenatal visits during pregnancy	No antenatal visits	40 (1.2)
	1 to 3	1213 (37.8)
	More than 3	1953 (60.9)
Place of delivery	Home	198 (6.2)
	Health facility	3007 (93.8)
Size of child at birth	Small	532 (16.6)
	Average	1668 (52.0)
	Large	1006 (31.4)
Had diarrhea in last two weeks	No	2727 (85.1)
	Yes	479 (14.9)
Had fever in last two weeks	No	2597 (81.0)
	Yes	609 (19.0)
Had cough in last two weeks	No	2292 (71.5)
	Yes	914 (28.5)
Vitamin A in last 6 weeks	No	685 (21.4)
	Yes	2521 (78.6)
Childes age in months	Infant	667 (20.8)
	12 to 59 month	2539 (79.2)

## Statistical analysis

### Logistic regression model

Bivariate binary logistic regression is a statistical model used to estimate the effect of predictors for binary outcome variables. In present study, let *Y*_1*i*_, *and Y*_2*i*_ are the dichotomies outcome of undernutrition and anemia of the *i*^*th*^ children under-five years, respectively. For dichotomies outcome *Y*_*ji*_ and a vector of independent variables X, bivariate binary logistic regression model is given by [36]:

$$\pi_{j(X)}=\frac{e^{\beta_{j0}+\beta_{j1}X_{1}+\beta_{j2}X_{2}+\cdots +\beta_{jp}X_{p}}}{{1+e}^{\beta_{j0}+\beta_{j1}X_{1}+\beta_{j2}X_{2}+\cdots +\beta_{jp}X_{p}}}=\frac{e^{X\beta_{j}}}{1+e^{X\beta_{j}}},j=1,2$$ (1)

Where πj(X)=P(Yji=1X), the probability of the *i*^*th*^ under-five children being stunted (*Y*_1*i*_), underweight (*Y*_2*i*_), and wasting (*Y*_3*i*_) given other predictors X. Consistently, the logit (log odds) that marked linear association with independent variables can be stated as:

logit[P(Yji=1X)]=βj0+βj1X1+βj2X2+⋯+βjpXp=Xβj,j=1,2 (2)

Odds ratio is the best method that is used to measure the relationship between categorical variables in the logistic regression model. It is the proportion of odds defined as:

ORj=πj(X1)1−πj(X1)πj(X2)1−πj(X2) (3)

To the best of our knowledge, no research has been conducted in Rwanda on the determinants of coexistence of both undernutrition and anemia using a bivariate binary logistic regression model; therefore, in this study, we will perform a separate analysis of stunting, underweight, wasting, and anemia in children under the age of five, as previously done by studies [37–40]. Using ordinal or binary logistic regression is adequate. Utilizing binary or ordinal logistic regression, however, misses the connection between anemia and starvation. To accomplish this, we take into account the link between anemia and undernutrition and, as a result, assess the unique effects of other factors. Bivariate logistic regression may therefore be a more practical choice. This statistical model is used to simultaneously model two binary outcome variables and assess how they relate to other factors [41, 42]. The marginal likelihoods can be modeled as a function of the explanatory variables. The model also assesses the relationship between anemia and undernutrition in children under the age of five. The data was initially imported and managed using SPSS version 27 software. Finally, the analysis was performed by R software version 4.0.5 using the VGAM package [43]. VGAM was familiarized with functions designed for fitting vector generalized linear and additive models.

### The goodness of fit test

An examination of the adequacy or goodness of fit of the model is compulsory before fitting. This can be accomplished by using the correct classification rate. The correct classification rate is the proportion of the number of correct predictions to the number of observations. The fitted model does a good job of estimating the data [44].

## Results

### Exploratory analysis

#### Characteristics of dependent variables

From a weighted total of 3,206 under-five children considered in this study, 1482 (46.2%) and 658 (20.5%) of the children suffered from anemia and undernutrition, respectively (see Table 2).

#### Characteristics of independent variables

The result of Table 3 showed that more than 25% of children under five years were obtained from the west (25.5%) and east (25.3%) regions of Rwanda. The majority of the participant’s families lived in rural areas (83.0%), while 35.9% of households had access to improved drinking water, and 85.7% of the households were led by a male household head. 20.8% of the children were infants, nearly half of the children (48.8%) were females, and 52.0% of the children had an average birth size. Most of the households (57.4%) have five to eight family members and more than half (55.5%) of the households have three or more under-five children. Mothers and their husbands who did attend higher education were 4.5% and 5.5%, respectively. Nearly one-fifth of all households (19.7%) have middle-class wealth index. Two weeks before the survey, 14.9, 19.0, and 28.5% of children had diarrhea, coughs, and fevers, respectively.

Table 4 shows the association of covariates with undernutrition and anemia using the chi-square (*χ*^2^) test. The result indicates that the source of drinking water, number of under-five children in the household, sex of the household head, age of the mother at first birth, birth order number of a child, and sex of a child were correlated with both anemia status and undernutrition status of under-five children in the present study.

**Table 4:** The association of predictors with child undernutrition and anemia (n = 3,206).

Predictors	Anemia status		P-value	Undernutrition status		P-value	
	No (%)	Yes (%)		No (%)	Yes (%)		
**Region**						
Kigali	262 (57.6)	192 (42.4)	0.002	376 (83.0)	78 (17.0)	0.009	
South	378 (61.0)	242 (39.0)		480 (77.3)	141 (22.7)		
West	425 (52.1)	391 (47.9)		625 (76.6)	191 (23.4)		
North	268 (53.1)	237 (46.9)		409 (81.2)	95 (18.8)		
East	483 (59.6)	328 (40.4)		665 (82.0)	146 (146)		
**Type of place of residence**						
Urban	326 (60.0)	217 (40.0)	0.079	464 (85.3)	80 (14 7)	0.000
Rural	1489 (55.9)	1174 (44.1)		2092 (78.6)	570 (21.4)	
**Source of drinking water**						
Not improved	1152 (56.0)	903 (44.0)	0.365	1634 (79.5)	420 (20.5)	0.725
Improved	664 (57.7)	487 (42.3)		922 (80.0)	230 (20.0)	
**Type of toilet facility**						
Not improved	466 (55.4)	375 (44.6)	0.413	616 (73.2)	226 (26.8)	0.000
Improved	1349 (57.0)	1016 (43.0)		1939 (82.0)	425 (18.0)	
**Number of household members**						
1 to 4	659 (55.1)	538 (44.9)	0.321	982 (82.0)	215 (18.0)	0.042
5 to 8	1062 (57.7)	777 (42.3)		1441 (78.4)	398 (21.6)	
More than 8	94 (55.3)	76 (44.7)		132 (78.1)	38 (21.9)	
**Number of children 5 and under in household**						
Only one	804 (58.3)	575 (41.7)	0.162	1115 (80.9)	264 (19.1)	0.384
Two	932 (55.8)	658 (44.2)		1175 (78.9)	315 (21.1)	
3 and more	179 (53.1)	158 (46.9)		266 (78.9)	71 (21.1)	
**Sex of household head**						
Male	1553 (56.7)	1194 (43.5)	0.827	2186 (79.6)	560 (20.4)	0.698
Female	262 (57.1)	197 (42.9)		369 (80.4)	91 (19.6)	
**Wealth index**						
Poor	722 (54,4)	604 (45.6)	0.068	983 (74.1)	343 (25.9)	0.000
Middle	357 (56.5)	275 (43.5)		483 (76.5)	149 (23.5)	
Riche	736 (59.0)	512 (41.0)		1089 (87.3)	159 (12.7)	
**Age of mother at 1st birth**						
Less than 20	565 (56.3)	438 (43.7)	0.321	780 (77.8)	222 (22.2)	0.198
20 to 34	1234 (56.9)	935 (43.1)		1747 (80.5)	422 (19.5)	
35 to 49	15 (44.1)	19 (55.9)		28 (82.4)	7 (17.6)	
**Body Mass Index of mother**						
Thin	60 (63.8)	34 (36.2)	0.013	58 (62.4)	35 (37.6)	0.000
Normal	1152 (54.8)	950 (45.2)		1609 (76.6)	492 (23.4)	
Overweight	444 (58.4)	316 (41.6)		658 (86.6)	102 (13.4)	
Obese	159 (63.6)	91 (36.4)		230 (92.0)	22 (8.0)	
**Anemia status of mother**						
No	1639 (58.0)	1188 (42.0)	0.000	2262 (80.0)	565 (20.0)	0.219
Yes	176 (46.4)	203 (53.6)		293 (77.3)	86 (22.7)	
**Covered by health insurance**						
No	357 (61.7)	222 (38.3)	0.007	416 (71.8)	163 (28.2)	0.000
Yes	1458 (55.5)	1169 (44.5)		2134 (81.4)	488 (18.6)	
**Highest education level of mother**						
No education	218 (56.6)	189 (46.4)	0.425	298 (73.2)	109 (26 8)	0.000
Primary	1189 (57.2)	891 (42.8)		1612 (77.5)	467 (22.5)	
Secondary	321 (55.8)	254 (44.2)		506 (88.0)	69 (12.0)	
Higher	87 (60.4)	57 (39.6)		139 (96.5)	6 (3.5)	
**Mothers’ husband education level**						
No education	219 (51.0)	210 (49.0)	0.074	306 (71.5)	122 (28.5)	0.000
Primary	1268 (57.8)	926 (42.2)		1725 (78.7)	468 (21.3)	
Secondary	227 (55.7)	181 (44.5)		358 (87.5)	51 (12.5)	
Higher	101 (57.7)	74 (42.3)		166 (94.9)	10 (5.1)	
**Current working status of mother**						
No	424 (51.4)	401 (48.6)	0.000	662 (80.3)	163 (19.7)	0.607
Yes	1391 (58.4)	990 (41.6)		1893 (79.5)	488 (20.8)	
**Birth order number of child**						
First	352 (56.5)	270 (43.5)	0.619	522 (84.1)	99 (15.9)	0.461
2 to3	771 (55.5)	619 (44.5)		1138 (81.9)	252 (18.1)	
4 to 5	416 (58.4)	296 (41.6)		542 (76.2)	169 (23.8)	
6 and more	276 (57.3)	206 (42.7)		353 (73.2)	131 (26.8)	
**Child is twin**						
Single birth	1778(57.1)	1338 (42.9)	0.003	2510 (80.6)	605 (19.4)	0.000
Multiple birth	37 (41.1)	53 (58.9)		45 (50.0)	46 (50.0)	
**Sex of child**						
Male	910 (55.5)	730 (44.5)	0.188	1299 (79.2)	342 (20.8)	0.461
Female	905 (57.8)	661 (42.1)		1256 (80.3)	309 (19.7)	
**Number of antenatal visits during pregnancy**						
No antenatal visits	21 (52.5)	19 (47.5)	0.000	30 (76.9)	9 (23.1)	0.162
1 to 3	632 (52.1)	581 (47.9)		947 (78.1)	267 (21.9)	
More than 3	1162 (59.5)	791 (40.5)		1578 (80.8)	375 (19.2)	
**Place of delivery**						
Home	116 (18.6)	82 (41.4)	0.563	134 (67.7)	65 (32.3)	0.000
Health facility	1699 (56.5)	1309 (43.5)		2421 (80.5)	586 (19.5)	
**Size of child at birth**						
Small	303 (56.8)	230 (43.2)	0.761	335 (62.9)	198 (37.1)	0.000
Average	935(56.1)	733(43.9)		1339 (80.3)	329 (19.7)	
Large	578 (57.5)	427 (42.5)		882 (87.8)	123 (12.2)	
**Had diarrhea in last two weeks**						
No	1564 (57.3)	1164 (42.7)	0.050	2214 (81.2)	513 (18.8)	0.000
Yes	251 (52.5)	227 (47.5)		342 (71.4)	137 (28.6)	
**Had fever in last two weeks**						
No	1500 (57.8)	1096 (42.2)	0.007	2094 (80.6)	503 (19.4)	0.008
Yes	316 (51.7)	294 (48.3)		462 (75.9)	147 (24.1)	
**Had cough in last two weeks**						
No	1311 (57.2)	981 (42.8)	0.289	1855 (80.9)	437 (19.1)	0.007
Yes	504 (55.1)	410 (44.9)		701 (76.7)	213 (23.3)	
**Vitamin A in last 6 months**						
No	192 (28.0)	493 (72.0)	0.000	563 (82.2)	122 (17.8)	0.070
Yes	1623 (64.4)	898 (35.6)		1993 (79.1)	528 (20.9)	
**Childes age in months**						
Infant	109 (16.3)	558 (83.7)	0.000	571 (85.6)	95 (14.4)	0.000
12 to59 months	1706 (67.2)	833 (32.8)		1984 (78.1)	555 (21.9)	

Table 5 displays the joint and marginal probabilities of anemia status and undernutrition status of under-five children and the odds ratio. The odds ratio (OR) is a usual measure that is used to define the relationship between the two dichotomous outcomes, and if the value of the odds ratio is one, it shows statistical independence [36]. In this result, the OR differed from unity (1.729). Hence, fitting the joint probability of child anemia and undernutrition status given a set of independent variables while considering the probable dependency between the two outcomes via a bivariate binary logistic regression model is very essential. Therefore, the bivariate logistic regression analysis of the anemia and undernutrition status of children with other predictors is presented in Table 6.

**Table 5:** Joint and marginal probability of child undernutrition and anemia.

		Undernutrition status		Marginal of anemia	Odds ratio (OR)
		Undernourished	Nourished		
Anemia status	Anemic	371 (0.116)	1111 (0.347)	1482 (0.462)	1.729
	Not anemic	280 (0.087)	1444 (0.450)	1724 (0.538)	
Marginal of undernutrition		651 (0.203)	2555 (0.797)	3206 (1.000)	

Key: Numbers in each cell is a frequency and its probability is in the parenthesis

**Table 6:** Parameter estimation of bivariate binary logistic regression modeling of child undernutrition and anemia.

Predictors	Anemia		Undernutrition	
	AOR (95% CI)	P-value	AOR (95% CI)	P-value
Intercept	1.24 (0.70,1.91)	0.089	1.43 (0.22,2.03)	0.203
Region				
Kigali	Ref.		Ref.	
South	0.965 (0.904, 1.030)	0.285	0.980 (0.932, 1.030)	0.423
West	1.067 (1.003,1.135)	0.041	0.990 (0.944,1.038)	0.678
North	1.059 (0.989,1.133)	0.100	0.969 (0.920, 1.021)	0.242
East	0.981 (0.920, 1.047)	0.569	0.968 (0.922,1.018)	0.203
Place of residence				
Urban	0.751 (0.573,0.984)	0.002	0.663 (0.456,0.965)	0.000
Rural	Ref.		Ref.	
Source of drinking water				
Not improved	Ref		Ref	
Improved	0.681 (0.466,0.996)	0.000	0.581 (0.338,0.998)	0.003
Types of toilet facility				
Not improved	Ref		Ref	
Improved	0.998 (0.960,1.038)	0.922	0.964 (0.935,0.993)	0.016
Family size				
1 to 4	Ref		Ref	
5 to 8	1.012 (0.968, 1.058)	0.608	1.012 (0.978,1.047)	0.496
More than 8	1.029 (0.938,1.130)	0.545	1.024 (0.953,1.100)	0.516
Number of under-five children in the household				
Only one	Ref		Ref	
Two	1.003 (0.967, 1.039)	0.889	1.001 (0.974,1.029)	0.954
Three and more	0.995 (0.935,1.058)	0.873	0.977 (0.932,1.024)	0.329
Sex of household head				
Male	Ref		Ref	
Female	0.984 (0.938,1.031)	0.489	0.988 (0.953,1.025)	0.523
Wealth index				
Poor	Ref		Ref	
Middle	0.988 (0.944,1.035)	0.612	0.999 (0.965,1.035)	0.969
Rich	0.976(0.930,1.024)	0.323	0.978 (0.943,1.015)	0.244
Age of mother at first birth				
Less than 20	Ref		Ref	
20 to 34	1.009(0.973, 1.046)	0.619	0.998 (0.971,1.026)	0.880
35 to 49	1.051 (0.896,1.234)	0.539	0.984 (0.870,1.113)	0.799
Body mass index of mother				
Thin	Ref		Ref	
Normal	1.063 (0.967,1.169)	0.202	0.884 (0.822, 0.950)	0.000
Overweight	1.021 (0.924,1.128)	0.688	0.849 (0.787,0.917)	0.000
Obese	1.004(0.896,1.124)	0.948	0.842 (0.772,0.919)	0.000
Anemia level of mother				
Not anemic	Ref		Ref	
Anemic	1.160 (1.104,1.218)	0.000	1.663 (1.242,2.225)	0.000
Covered by health insurance				
No	Ref		Ref	
Yes	1.043 (0.997,1.090)	0.065	0.971 (0.939,1.005)	0.097
Highest education level of mother				
No education	Ref		Ref	
Primary	0.959(0.911,1.009)	0.107	0.982 (0.944,1.021)	0.359
Secondary	0.944 (0.881,1.012)	0.104	0.949 (0.900,1.001)	0.052
Higher	0.868 (0.777,0.971)	0.013	0.943 (0.865,1.027)	0.179
Highest education level of mothers husband				
No education	Ref		Ref	
Primary	0.948 (0.902,0.995)	0.031	0.982 (0.945,1.019)	0.331
Secondary	1.005(0.937,1.078)	0.884	0.972 (0.21,1.026)	0.309
Higher	1.024 (0.922,1.137)	0.657	0.972 (0,897,1.053)	0.485
Current working status of mother				
No	Ref			
Yes	0.959 (0.923,0.997)	0.034	0.993 (0.964,1.023)	0.629
Birth order number				
First	Ref		Ref	
2 to 3	0.999(0.952,1.049)	0.984	1.010 (0.973,1.048)	0.606
4 to 5	0.949 (0.890,1.011)	0.103	1.035 (0.986,1.087)	0.161
6 and more	0.949 (0.882,1.022)	0.165	1.036 (0.979,1.096)	0.218
Child is twin				
Single	Ref		Ref	
Multiple	1.727 (1.292, 2.310)	0.000	1.278 (1.183,1.380)	0.000
Sex of child				
Male	Ref		Ref	
Female	0.985 (0.955, 1.017)	0.367	0.989 (0.965,1.014)	0.379
Number of antenatal visits during pregnancy				
No visit	Ref		Ref	
1 to 3	1.034 (0.880,1.214)	0.683	1.048 (0.926,1.185)	0.459
More than 3	1.002 (0.853,1.177)	0.980	1.046 (0.924,1.185)	0.479
Place of delivery				
Home	Ref		Ref	
Health facility	0.998 (0.931,1.070)	0.959	0.951 (0.902,1.004)	0.067
Size of children at birth				
Small	Ref		Ref	
Average	0.567 (0.462,0.696)	0.000	0.856 (0.827,0.886)	0.000
Large	0.823 (0.700,0.968)	0.000	0.822 (0.791,0.853)	0.000
Had diarrhea in the last two weeks				
No	Ref		Ref	
Yes	1.134 (1.210, 2.792)	0.000	1.052 (1.015,1.091)	0.006
Had fever in the last two weeks				
No	Ref		Ref	
Yes	1.076 (1.028,1.128)	0.001	1.152 (1.312, 2.981)	0.000
Had cough in the last two weeks				
No	Ref		Ref	
Yes	0.974 (0.936,1.013)	0.912	1.007 (0.976,1.038)	0.676
Take vitamin A in the last six months				
No	Ref		Ref	
Yes	0.870 (0.832,0.910)	0.000	0.986 (0.952,1.020)	0.415
Children age in months				
Infant	Ref		Ref	
12 to 59 months	1.697 (1.184, 2.433)	0.000	1.054 (1.017,1.092)	0.003

AOR = Adjusted odds ratio; CI: Confidence interval; Ref: reference category

### Parameter estimates

Table 6 displays the relationship between predictors and the undernutrition and anemia status of children under the age of five while taking into account the interdependence between the two conditions. An odds ratio (OR) of 1.729 was used to calculate the relationship between child undernutrition and child anemia; a number that differs from one denotes a significant relationship between the two conditions. The impact of each predictor on a child’s undernutrition and anemia was assessed after the association between child undernutrition and child anemia was examined. The results of this study thus showed that region, mother’s highest educational level, husband’s highest educational level, mother’s present employment status, and a child consuming vitamin A during the previous six months were statistically associated with children’s anemia. The type of toilet facility and the mother’s BMI were statistically associated with undernutrition in children under five. Whereas, according to the result, both childhood undernutrition and anemia are related to a child’s age, place of residence, drinking water source, anemia level of the mother, child if a twin, birth size of the children, diarrhea, and fever. The ratio of the number of correct predictions to the number of observations, i.e., the correct classification rate (CCR), is 88.8%, which is very high. This indicates that the estimated model was a good fit for the data.

## Discussion

By assuming a relationship between child anemia and undernutrition, this finding sets out to show the impact of children’s, maternal, household and community factors on undernutrition and anemia in children. Due to the strong association between the three undernutrition indicators, a single composite index measure for stunting, wasting, and underweight was created using principal component analysis [27, 28, 45]. The bivariate binary logistic regression model and exploratory data analysis were used to find a significant association between child undernutrition and child anemia. Given the mother, household, and community characteristics of under-five children, the data imply that undernutrition in children and anemia in children are significantly associated. This finding is consistent with research from Ghana [46], Ethiopia [47], and Bangladesh [48]. A possible defense is that since poor nutrition is linked to ill health, pollution and invasions also have synergistic effects on the prevalence of anemia and micronutrient deficiencies. Furthermore, malnourished kids are more prone to micronutrient deficiencies such as those in iron, vitamin A, vitamin B12, and folic acid, which are necessary for hemoglobin and DNA fusion during the production of red blood cells and, subsequently, the effects of anemia [49]. Malnutrition and anemia typically have similar root causes, and it is possible that several forms of malnutrition will coexist in comparable children and work synergistically to induce anemia to develop. Additionally, the intestinal epithelium of malnourished children may suffer, decreasing absorption and aiding in the growth and decline of anemia [50]. Therefore, low hemoglobin levels may also interfere with the direct development of childhood.

The prevalence of undernutrition and anemia was found to be high (20.2% and 43.2%, respectively). This result is consistent with previous research [48, 51]. The high prevalence of childhood anemia and undernutrition may be explained by population growth [5]. Region was found to be a significant contributor to childhood anemia in our study, which is consistent with other findings in Rwanda [52, 53]. In comparison to children born in Kigali, those born in the West are more likely to develop anemia. According to the RDHS 2019/20, this can be the result of greater malaria prevalence rates in the western area than in Kigali City. Additionally, compared to women in other regions of the nation, women in Kigali City may have easier access to information on maternity healthcare as well as medical facilities and services [5]. The other justification is that the Western Province, which has over 35% of its households categorized as having food insecurity, 42 percent of all severely food-insecure households in Rwanda are located in the Western Province, although this only accounts for 22% of all households in the nation [54]. As a result, food insecurity levels elevated the risk of anemia because, in addition to having insufficient intakes of crucial micronutrients, they also had greater access to and intakes of dietary components that interfere with the absorption of minerals, which can cause anemia [55].

In this study, maternal education was identified as a possible predictor of anemia. Compared to children with higher maternal education, those with lower maternal education are more likely to have anemia that is worse. This conclusion was corroborated by a number of earlier studies from Ethiopia, Rwanda, Togo, Nigeria, Sub-Saharan Africa, and Togo that showed children with more educated moms were less likely to be anemic than children with less or no schooling [8, 33, 53, 56–58]. This shows that raising maternal literacy levels is crucial for lowering the incidence of childhood anemia. This may be the case because a more educated mother is more likely to be aware of the need for good nutrition and access to quality healthcare, both of which can help prevent anemia in children [27, 59]. Additionally, a woman with education can easily earn more money than a mother without education, which improves the household’s standard of living [8]. Children with primary school-educated parents were less likely to be anemic than those with uneducated parents. According to other studies [60], parents with formal education are more educated about good child cleanliness and feeding habits, which can reduce childhood anemia [58]. This might be the case because educated parents are more knowledgeable about diet and health than uneducated parents are, and they also employ child-rearing techniques that promote the health of their children [61].

This result indicates a substantial connection between vitamin A supplementation and childhood anemia. When compared to children who did not take vitamin A supplements in the six months prior to the survey, children who did take vitamin A supplements prior to the survey had a lower risk of developing anemia. This is most likely because vitamin A helps to ensure that hematologic and linear growth are optimal [22, 51, 62]. Vitamin A also reduces the incidence of infection-related anemia by enhancing both humoral and cell-mediated immunity [63]. The prevention of stunting and promotion of child growth are additional advantages of vitamin A supplementation [51, 64], it is evident that under-five children who are vitamin A deficient are more likely to experience anemia and stunting [51]. This discovery backs up earlier studies [8, 65]. Children who reside in homes with inadequate toilet facilities are more likely to experience malnutrition than children who reside in homes with appropriate toilet facilities. Poor toilet hygiene can increase mother-child-environment interactions, exposing children to more illnesses as well as other opportunistic infections (diarrhea, fever) brought on by tainted food, water, or the environment. These are recognized reasons why children grow more slowly [66]. This result backs up earlier studies from Bangladesh and Cameroon [67, 68].

This study shows that a child’s age significantly affects their nutritional and anemia status. Both undernutrition and anemia were more likely to occur in infants than in children between the ages of 12 and 59 months. The risk of malnutrition and anemia rose with age, according to studies from Angola, Cameroon, India, Lesotho, Ethiopia, and Bangladesh [1, 48, 51, 67, 69, 70]. This finding is in line with those studies. This is a result of the late introduction of supplementary foods with inadequate nutritional quality [71]. This suggests that it is crucial to start supplementary feeding properly and on time in order to address the children’s expanding nutritional needs because undernutrition in children rises with age [72].

A rural child was more likely than an urban under-five child to be undernourished and anemic, per research [18, 42, 60, 73]. This might be a result of the inadequate care that rural children receive in medical facilities, their nutritional requirements, and the absence of other infrastructure. Parents of children in rural locations often lack the necessary knowledge to properly raise their offspring. Even basic needs like food, shelter, clothing, and medical care are in short supply. The majority of young children under five enter and leave the house naked, which exposes them to madness and other potentially dangerous substances [42].

Because low birth size is thought to be an indicator of restricted intrauterine growth [74], perceived child size at birth has a significant impact on the child’s nutritional and anemia status [75]. Children born with smaller-than-average or large birth sizes were more likely to be malnourished and anemic, according to our findings. These findings were consistent with previous research, which found that low birth weight children are significantly more likely to be undernourished and anemic later in life due to inadequate fetal nutrition, and low birth weight has been linked to a variety of poor health and nutritional outcomes [76, 77].

In this study, children who had diarrhea and fever had a higher risk of developing undernutrition and anemia than children who did not have diarrhea or fever. It agrees with studies from Ecuador and India [70, 78]. This could be because children suffering from febrile and diarrheal illnesses may experience a loss of appetite as well as decreased absorption of essential nutrients (iron, folate, and vitamin B12), which may increase the likelihood of anemia [79]. Furthermore, diarrhea and fever may indicate the presence of infectious diseases like visceral leishmaniasis, malaria, hookworm, ascariasis, giardiasis, and amoebiasis, which are the leading causes of anemia in children [79, 80]. Several studies have confirmed the link between diarrhea, fever, and malnutrition, resulting in a vicious cycle [81, 82]. Intestinal infections suppress appetite, increase catabolism, and impair intestinal absorption of nutrients from food, making the underweight more vulnerable to severe malnutrition [83, 84]. As a result, child malnutrition causes immune dysfunction, such as impaired cell-mediated immunity, cytokine and immunoglobulin production, lowering immunity and predisposing a child to infectious diseases [81, 82]. Thus, an unhealthy environment is frequently caused by diarrhea and fever [85].

Children with multiple birth types were more likely than single-ton children to have severe undernutrition and anemia, which is consistent with the conclusion of [51]. Premature births and low birth weights are more probable in multiple pregnancies, which increases competition for nutrition and anemia [39]. In comparison to children from families with an improved water source, children from families without an improved water source were more likely to have worse undernutrition and anemia, which is consistent with prior research [67]. The source of drinking water was also a significant factor in undernutrition and anemia. By lowering parasite infections, diarrheal illnesses, and environmental intestinal dysfunction, improved water and sanitation can affect anemia and undernutrition [86].

Finally, maternal anemia significantly enhanced the likelihood of child anemia and undernutrition, just like in earlier research [70, 74]. Children of anemic mothers are more likely to be undernourished, according to the interaction between undernutrition and maternal anemia status [48, 87]. The fact that several anemia-related variables are shared by both anemia types may help to explain the high correlation between maternal and child anemia [8]. For instance, the mother and child may have access to the same iron-rich micronutrient food source and adhere to a comparable eating pattern. They also live in the same environment, have access to the same medical services, and perhaps share some genetic characteristics [88].

## Strength and limitation of the study

The study’s strengths include meaningful, high-quality data on children’s health, homes, and communities, as well as data from a nationally representative population-based study. The study’s huge sample size and random selection of participants from throughout the nation also enable findings to be extrapolated to kids between the ages of 6 and 59 months. Additionally, whether employing binary or ordinal logistic regression, the relationship between stunting, underweight, and wasting is disregarded. Multivariate logistic regression might be a preferable choice in this situation. This statistical model is used to simulate two or more binary outcome variables simultaneously and assess their relationship to other predictors. It satisfies the requirements for modeling marginal likelihood as a function of explanatory variables. Simultaneously, the model looks into the link between stunting, being underweight, and wasting in children under the age of five. The main limitation of this study is that the demographic health survey datasets are prone to errors, resulting in the inability to measure causal effects due to their cross-sectional nature.

## Conclusion

In conclusion, this study found a concerning high level of co-morbid anemia and stunting among Rwandan children, which was linked to a variety of dietary and non-dietary factors originating at the household, community, maternal, and child levels. The high prevalence of anemia and undernutrition at the same time suggests that both burdens must be addressed concurrently. Strengthening existing comprehensive public health and nutrition interventions, with a focus on the multifactorial nature of both undernutrition and anemia, could be an important consideration in reducing the burden of undernutrition and anemia in Rwanda and elsewhere.

## Abbreviations

DHS: Demographic health survey
EAs: Enumeration areas
RDHS: Rwanda demographic and health survey
Ref: Reference Category
HAZ: Height for age standardized score
RPHC: Rwanda Population and Housing Census
WAZ: Weight for age standardized score
WHZ: Weight for height standardized score
OR: Odds ratio
VGAM: Vector Generalized Additive Model
